# The Response of More Health Focused and Less Health Focused People to a Physical Activity Calorie Equivalent Label on Discretionary Snack Foods

**DOI:** 10.3390/nu11030525

**Published:** 2019-02-28

**Authors:** Claudia Hartley, Russell SJ Keast, Djin Gie Liem

**Affiliations:** Deakin University, Centre For Advanced Sensory Science, School of Exercise and Nutrition Sciences, Burwood Highway, Burwood, VIC 3125, Australia; c.hartley@deakin.edu.au (C.H.); russell.keast@deakin.edu.au (R.SJ.K.)

**Keywords:** discretionary snack foods, PACE labelling, liking, consumption, health focus, labelling, health consciousness

## Abstract

A Physical Activity Calorie Equivalent (PACE) label shows the minutes of physical activity required to burn off the caloric content of a particular food. This study investigated the influence of PACE labelling on liking and consumption of discretionary snack foods in a group of more health focused and less health focused consumers. Participants (*n* = 97) tasted and rated (i.e., liking, prospective consumption) a range of snack foods with or without a PACE label. Total sampling consumption was also measured. Participants completed a shortened version of the International Physical Activity Questionnaire and the General Health Interest Scale questionnaire. Paired samples *t*-test, independent samples *t*-tests, a General Linear Model and Chi-Square tests were used to check for statistical significance. For more health focused participants (*n* = 57), the PACE label decreased only liking (*p* = 0.02). The PACE label was not effective in reducing liking (*p* = 0.49), prospective consumption (defined as the amount of the sample participants thought that they could consume) (*p* = 0.10) or consumption (*p* = 0.41) of energy-dense discretionary snack foods for less health focused individuals (*n* = 40). The level of participants’ physical activity did not facilitate the influence of PACE labelling on liking, consumption or prospective consumption. The PACE label was found to not be effective among less health focused individuals or the overall sample population. Therefore, the PACE label may not be an effective labelling strategy to reduce the liking or consumption of discretionary snack foods.

## 1. Introduction

According to the World Health Organisation, overweight in Caucasian populations is defined as having a body mass index (BMI) of greater than or equal to 25 kg/m^2^ and less than 30 kg/m^2^ and obesity as having a BMI of greater than or equal to 30 kg/m^2^ [1]. During the last two decades in the Australian population, BMI has been increasing steadily [2]. In Australia, 63% of adults are currently overweight or obese and this is a 10% increase since 1995 [3]. Furthermore, one in four Australian children are obese or overweight [4]. Overweight and obesity are the second highest contributor to burden of disease in Australia [3]. The increasing prevalence of obesity is linked to the development of chronic diseases such as hypertension, type 2 diabetes, coronary heart disease, depression, elevated cholesterol levels and musculoskeletal disorders [2]. In Australia in 2008, it was estimated that the cost of overweight and obesity was $58.2 billion [5]. This includes direct health costs, carer costs and productivity losses [5]. This is in contrast to an estimated figure of $21 billion in 2005 [6].

Obesity is a result of continuously consuming a larger amount of energy than what is used by the body [7,8,9,10]. About one third (35%) of daily energy consumed is derived from discretionary foods [7]. The Australian Dietary Guidelines define discretionary foods as foods and drinks that do not provide necessary nutrients and tend to be high in saturated fats, sugars and salt. They are therefore described as energy-dense and are high contributors to excess energy intake [7,11]. About 35% of energy derived from discretionary food comes from foods which can be classed as discretionary snack foods [7]. Discretionary snack foods specifically include frozen milk products such as ice-cream, sweet biscuits, cakes, muffins and other cake-like desserts, chocolate, confectionery and savoury snack foods which are responsible for 10.5% of total daily energy intake [7,12]. 

In this paper, discretionary snack foods are defined as energy-dense, processed, commercially available nutrient-poor foods, which are eaten outside of main meals. A similar definition has been used by Niven et al. [12]. Many commercially available discretionary snack foods are energy-dense, processed and act as major contributors to calorie and saturated fat intake [13,14,15]. 

The consumption of discretionary snack foods is positively correlated with weight gain [15,16]. This is partly due to humans having a weak ability to recognize energy-dense foods such as discretionary snack foods and are unable to appropriately decrease the consumption of those foods to maintain an energy balance [17]. Several studies have shown that a decrease in the consumption of energy-dense foods such as discretionary snack foods may help to promote weight loss [18,19]. Furthermore, a consistent positive energy imbalance of 209 kJs could lead to a weight gain of 5 pounds or 2.2 kg per year [20]. As most discretionary snack foods are energy-dense, a small dietary change to reduce the intake of these foods would have effective health outcomes and provides a strong rational for the implementation of various strategies to reduce excess energy intake [15].

Currently, several strategies exist to decrease the consumption of discretionary snack foods. One strategy is for manufacturers to reformulate products to make them less energy-dense. This strategy, however may not be effective overall and is not popular with consumers as they feel their choice is being taken away [21]. An alternative strategy that has been implemented to decrease the consumption of energy-dense discretionary snack foods is front of pack (FOP) labelling. FOP labelling in this context refers to nutrition FOP labelling which includes messages about the nutritional value of the product displayed on the front of the pack. This may be a preferred strategy to product reformulation as FOP labelling provides consumers with nutritional information and allows them to choose for themselves if they wish to consume the product or not. FOP labelling includes labelling initiatives such as calorie content labelling [22], the Health Star Rating system [23] and the Traffic Light system [23]. FOP labelling has been found to influence a consumers’ product choice, liking and also consumption [23,24,25]. As FOP labelling is designed to be simple and straightforward, consumers notice this type of labelling more quickly and more often than other types of labelling such as back of pack labelling [26]. However conflicting research exists on how effective these existing labelling systems (Health Star Rating system, the Traffic Light system and calorie content labelling) are and consumers generally find them confusing [22,23,27,28]. This confusion is partially due to poor consumer knowledge concerning calorie intake [9,22] and a large portion of the population is unaware of the number of calories that constitutes a healthy weight [9,22]. In addition, systems like the Health Star Rating system have been criticised for their inability to clearly communicate to consumers the promotion of foods from the core food groups while discouraging discretionary foods [29]. There is therefore a need for a new labelling strategy for discretionary snack foods that is simple, straightforward and easy to interpret for all consumers. 

An alternative strategy to decrease energy consumption from discretionary snack foods is to implement Physical Activity Calorie Equivalent (PACE) labelling. This type of labelling is a visual representation of the amount of physical activity required to burn off the caloric content of a particular food [30,31]. It is thought that the PACE label is more relevant and easier to understand than simply stating the calories [32,33]. There is promising evidence that supports the use of PACE labelling on pre-packaged foods such as discretionary snack foods to encourage lower energy food choice [31]. PACE labelling is well liked by consumers [8] and is effective in consistently decreasing calorie consumption [30]. PACE labelling has also been found to successfully encourage physical activity among consumers [30]. A recent study suggests that PACE labelling decreases the consumption and prospective consumption of snack foods amongst nutrition students [34]. It remains however unclear if similar effects can be obtained with a more representative sample of the population which includes more health focused and less health focused people. Being less health focused can be seen as a determinant of an unhealthy lifestyle [35]. Compared to more health focused consumers, less health focused consumers are concerned less about their health or wellness and are in general, less motivated to improve or retain their health and overall quality of life to reduce the risk of disease by engaging in healthy behaviour and being self-conscious about their health [36]. Less health focused consumers show a lower engagement in healthy behaviours and are less self-conscious about their health [37,38,39]. Research has also found that less health focused consumers look at FOP labelling less than consumers who are more health focused [40]. Therefore, to investigate the influence of FOP labelling on food perception and consumption, it is important to include less health focused consumers. 

This paper will examine one particular way of decreasing energy consumption through the use of PACE labelling and how segments of consumers (i.e., less health focused and more health focused) respond differently to this label. 

## 2. Materials and Methods

### 2.1. Overview

In this study, participants tasted and rated (i.e., their liking and prospective consumption) three snack foods. The PACE label was assigned to samples in a balanced randomised fashion. After tasting and rating, sampling consumption was measured and participants were given three questionnaires, which measured general demographics, health focus and perceived physical activity level. Participants also rated their hunger prior to testing. Participants were tested in the sensory laboratory at The Centre for Advanced Sensory Science (CASS) at Deakin University.

### 2.2. Participants

To calculate the number of participants required, power analyses based on a linear multiple regression with an assumed small to medium effect size (f^2^ = 0.15) [34] were used. These suggested that a total sample size of *n* = 71 would provide enough power to detect differences in liking and/or consumption. Participants were recruited either through handing out flyers at Deakin University or through an existing consumer database (CASS Participant Database) at Deakin University, which contains a representative sample of 18 to 65 year olds living in mostly the eastern suburbs of Melbourne. Participants who were under 18 years of age, had food intolerances or allergies and/or were not proficient in English were excluded. After testing, 3 participants were excluded from the data set due to errors in their data. In total, 97 participants completed the study.

### 2.3. Materials

**Figure 1:**
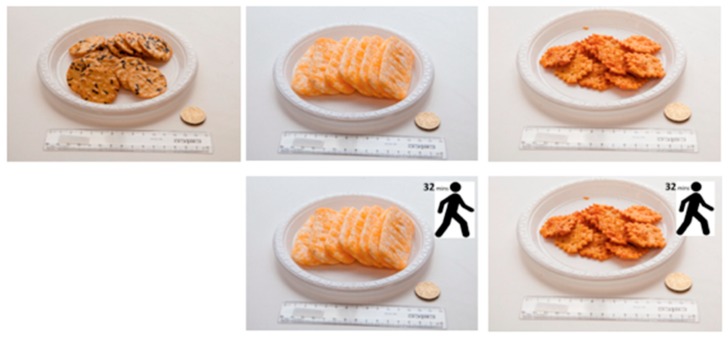
Examples of the snack foods (L-R: Sakata Seaweed Rice Cracker, Want Want Cheese Rice Cracker, Arnott’s Nacho Cheese Shapes) without and with the PACE label.

In a pilot study, 24 snack foods were trialled by five members of CASS. From this group, two unfamiliar (i.e., Want Want Cheese Rice Crackers, Taiwan and Shrimp Peanut Crackers, Thailand—both purchased at Asian specialty shops), two commonly available snack foods (i.e., Arnott’s Nacho Cheese Shapes, Australia and Honey Cashew Nuts, Australia) and one commonly available control snack food (Sakata Seaweed Rice Crackers, Australia) were selected and subsequently used by Hartley et al [34]. Hartley’s study revealed that the Shrimp Peanut Crackers had low consumption rates and the Honey Cashew Nuts had very high liking ratings which could result in a potential ceiling effect. Therefore for this study, three moderately liked snack foods (one familiar snack food (Arnott’s Nacho Cheese Shapes), one unfamiliar snack food (Want Want Cheese Rice Crackers) and one control snack food (Sakata Seaweed Rice Crackers) from the study by Hartley were chosen (Figure 1). To make both the familiar and unfamiliar samples more comparable, two cheese flavoured snack foods were chosen as they had similar flavour profiles. The samples were coded with a blinded 3-digit code and each sample code can be seen below (Table 1). Twenty-five grams (1 serving size) of each sample was weighed out prior to testing and presented on a paper plate and napkin.

### 2.4. Questionnaires

Three questionnaires were used in this study. A general demographics questionnaire was used to gather participant’s information about their age and height. To measure the participants’ level of health focus, participants completed a health focus questionnaire which was based on the General Health Interest Scale developed by Roininen [41]. This scale was developed as a 7 point Likert-type scale (responses ranging from strongly disagree, somewhat disagree, disagree, neither agree nor disagree, agree, somewhat agree and strongly agree) to measure the participant’s perceived importance of healthiness [41] (see Table 2). The General Health Interest Scale has been previously utilised by other studies [42,43] as a valid method with both external and internal validity [41]. Previous research suggests that a high score on this scale is associated with healthier food choices [41]. To calculate the health focus score for each participant, each score was added together to give an overall score. Four statements were negatively phrased (as indicated by ‘R’ below in Table 2) and were recoded. The summated scores were split using the median value and those who scored above this value were considered to be more health focused. Those who scored below this value were considered to be less health focused. 

Participants also completed a shortened version of the International Physical Activity Questionnaire (IPAQ) to determine their physical activity levels [44]. The participant’s physical activity was calculated into weekly MET (Metabolic Equivalent Value)—minutes using the appropriate formula [44,45].

Total MET − minutes/week = (Walking METs × min × days) + (Moderate METs × min × days) + (Vigorous METs × min × days)(1)

To calculate physical activity levels, each participant’s scores for physical activity corresponded to a range of minutes (i.e., 1 = 0 min, 2 = 0–15 min, 3 = 16–30 min, 4 = 31–45 min etc.). The middle number from each minute range was then calculated to give an amount of physical activity completed. To calculate physical activity levels, the new value for minutes of vigorous and moderate physical activity and walking were multiplied by the frequency completed per week in days. This was then multiplied by the corresponding MET value which were outlined by Craig et al [44] (Vigorous activity = 8 METs, moderate activity = 4 METs, moderate walking = 3.3 METs). These values were added together to provide total MET minutes per week for each participant. Participants who completed at least 600 MET-minutes per week were deemed to be physically active. Participant who completed less than 600 MET-minutes per week were deemed to be physically inactive. This was in accordance with the physical activity guidelines [46].

### 2.5. Procedure

Participants sat at their own partitioned cubicle fitted with a computer, tap, sink and serving hutch. Participants were instructed to read and sign a Plain Language Statement and Consent Form. The researcher used a script to explain the testing session to the participants. The script was as follows, “Today you will be presented with a number of snack foods. These snack foods will also be shown on a photo on your screen. In each photo you might also see a pictogram of a man walking. This represents the number of minutes you would need to walk to burn the amount of snack food that you are presented. You can eat as much of the snack foods as you want. Please do not overthink your answers and just provide the answer that first comes to mind. When you are ready for your first sample, please turn on your light switch. When you are finished with that sample, please turn your light switch on again. There is a 30-s break between each sample and in that time, you may eat the crackers and drink the water provided.”. A script was used so that all participants were told the same information across all the sessions. Participants then started the tasting session.

**Figure 2:**
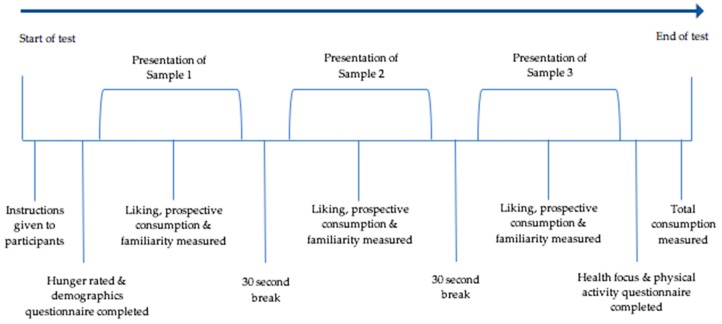
The procedure of a testing session.

Participants first completed the demographic questionnaire and were prompted to also rate their level of hunger. This was rated on a 5 point Likert-type scale (1 = not hungry at all, 2 = a little bit hungry, 3 = hungry, 4 = very hungry, 5 = extremely hungry). The participants were then presented with 1 serving size (25 grams) of each of the 3 samples; a control sample (Sakata Seaweed Rice Crackers), a familiar sample (Arnott’s Nacho Cheese Shapes) and an unfamiliar sample (Want Want Cheese Crackers). The control sample was always presented first to minimise first order bias. The familiar and unfamiliar samples were presented either with or without a PACE label. The sample order was done using balanced randomisation. Upon receiving each sample, participants were asked to rate their familiarity, liking and prospective consumption of each sample. Familiarity was measured on a 5-point Likert-type scale (1 = not at all familiar, 2 = not familiar, 3 = somewhat familiar, 4 = familiar, 5 = very familiar). Liking was measured on a 9-point hedonic scale (1 = dislike extremely, 2 = dislike very much, 3 = dislike moderately etc.) and prospective consumption was measured on a 9-point scale with each answer increasing in half gram serving increments (1 = none, 2 = ½ a serving or less, 3 = 1 serving, 4 = 1.5 servings etc.). A picture of each sample was presented on the participant’s computer screen either with or without a PACE label. In between each sample, participants had a 30 s break where they were instructed to drink water and eat the crackers provided. After each participant received the 3 samples, they completed a physical activity questionnaire and a health focus questionnaire. Upon completion of the testing session, participants were compensated for their time and given a $5 Woolworths Gift Card. The samples then were weighed again after testing to gain sampling consumption information. An overview of a testing session can be seen below in Figure 2. All data was collected using Compusense Cloud software as part of the Compusense Academic Consortium (Compusense Inc.).

### 2.6. Statistical Analyses

IBM SPSS Statistics version 24 was used for all analyses. The demographic information of participants such as age, height and gender, as well as liking scores, prospective consumption scores and total consumption (in grams) were described using means and standard deviations. Percentages were used to describe categorical variables such as gender and physical activity. 

Paired samples *t*-tests were used to examine any differences between the PACE label and liking, prospective consumption and consumption for familiar and unfamiliar products. Independent samples *t*-tests were performed to examine any differences between the PACE label and liking, prospective consumption and consumption for familiar and unfamiliar products for both more health focused and less health focused participants and physically active and physically inactive participants. A Chi-Square test was used to examine any possible associations between being health focused and physically active and to compare categorical variables such as age and physical activity groups (e.g., physically active vs inactive). A significance value of *p* < 0.05 was deemed to be statistically significant. Values are represented as means and standard deviation. 

In order to investigate the potential interaction effect between gender and PACE labelling on liking, a General Linear model was applied. In this model, liking was selected as the dependent variable and gender and PACE labelling (PACE vs no PACE) were selected as the fixed variables. The General Linear Model was repeated with either prospective consumption or consumption as the dependent variable. In order to investigate the main effects of PACE labelling and health focus, and the interaction between PACE labelling and health focus on liking, a General Linear Model was applied. In this model “liking” was selected as the dependent variable, and PACE labelling (PACE vs no PACE) and health focus (more health focused vs. less health focused) were selected as the fixed variables. To investigate the influence of BMI on this model, BMI was entered as co-variate in a separate General Linear Model. The General Linear Models were repeated with either prospective consumption or consumption as the dependent variable. To investigate the main effects of, PACE labelling and physical activity and the interaction between PACE labelling and physical activity on liking, a General Linear Model was applied. In this model “liking” was selected as the dependent variable, and PACE labelling (PACE vs. no PACE) and physical activity (physically active vs physically inactive) were selected as the fixed variables. The General Linear Model was repeated with either prospective consumption or consumption as the dependent variable.

### 2.7. Ethics

Ethics approval for this study was granted by the Deakin University Human Advisory Group and assigned the ethics number HEAG-H 50_2017. This study was conducted in accordance with the Declaration of Helsinki. All subjects gave their informed consent for inclusion before they participated in the study.

## 3. Results

### 3.1. Demographic Information

A total of 34 and 63 females (35.3 years ± 13.2) completed the study. It was found that 41% (*n* = 40) of participants were classed as less health focused and 59% (*n* = 57) were classed as more health focused. Approximately four fifths (80.4%) of participants were classed as physically active and about one fifth (19.6%) were classed as physically inactive. A summary of the demographic information of participants is presented below in Table 3. More health focused participants were more likely to be female, more likely to be physically active and had a lower weight and BMI (all *p*-values <0.001).

### 3.2. Liking

**Figure 3:**
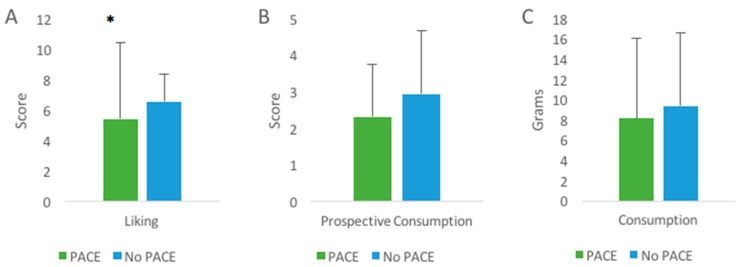
The impact of a PACE label on health focused participants. The PACE label decreased liking in more health focused participants significantly (*p* = 0.02) (panel **A**). Prospective consumption (panel **B** (1 = 1 serving = 25 grams, 2 = 2 servings = 50 grams etc.)) and consumption (panel **C** (consumption was measured in the amount (in grams) that participants sampled)) were not significant. Significant values are displayed: * *p* < 0.05. Error bars are shown as standard deviation.

For all products, the average liking score was 6.2 ± 2.0 on a 9-point hedonic scale. Overall, familiar products were liked more than unfamiliar products independent of PACE labelling (*p* = 0.00) (6.7 ± 1.7 vs. 5.5 ± 2.4 respectively). Gender did not play a significant role in the influence of the PACE label on liking (interaction effect PACE labelling × Gender *p* = 0.80), prospective consumption (interaction effect PACE labelling × Gender *p* = 0.90), or consumption (interaction effect PACE labelling × Gender *p* = 0.74). The overall model with PACE labelling and health focus as fixed factors and liking as the dependent variable was statistically significant (*p* = 0.02). The results show that although there was no main effect of PACE labelling (*p* = 0.18), or health focus (*p* = 0.33) on liking, there was a significant interaction effect of PACE labelling and health focus on liking (*p* = 0.02). For more health focused participants, the PACE label decreased liking in comparison to no PACE label significantly (5.4 ± 2.5 vs. 6.6 ± 1.8, *p* = 0.02 respectively) (Figure 3 panel A). For less health focused participants, the results for liking were not significant (*p* = 0.49). For familiar products, independent of a PACE label, less health focused participants had higher levels of liking in comparison to unfamiliar products (*p* = 0.02) (6.8 ± 1.7 vs. 5.8 ± 2.2 respectively). When also looking at more health focused participants, familiar products were liked more than unfamiliar products (*p* = 0.001) (6.6 ± 1.7 vs. 5.3 ± 2.6 respectively). When BMI was included as co-variate, the model was no longer significant (*p* = 0.45). However, BMI itself was not correlated to liking (r = −0.02, *p* = 0.09).

The overall model with PACE labelling and physical activity as fixed factors and liking as the dependent variable was not statistically significant (*p* = 0.34). When looking at physically inactive participants independent of a PACE label, familiar products had higher liking than unfamiliar products (*p* = 0.00) (7.3 ± 1.1 vs. 5.3 ± 2.4 respectively). Physically active participants also had significantly higher liking for familiar products in comparison to unfamiliar products (*p* = 0.01) (6.5 ± 1.7 vs. 5.6 ± 2.4 respectively). 

### 3.3. Prospective Consumption

For all products, the average prospective consumption score was 2.9 ± 1.9. Familiar products were scored higher for prospective consumption in comparison to unfamiliar products independent of a PACE label (*p* = 0.01) (3.3 ± 1.9 vs. 2.6 ± 1.8 respectively). The overall model with PACE labelling and health focus as fixed factors and prospective consumption as the dependent variable was statistically significant (*p* = 0.01). The results show that there was no main effect of PACE labelling (*p* = 0.40). There was a main effect of health focus on prospective consumption (*p* = 0.01). That is, more health focused participants significantly expected to eat less snack foods compared to less health focused participants. There was no interaction effect between PACE labelling and health focus on prospective consumption (p=0.14). Health focused participants had higher prospective consumption for familiar products in comparison to unfamiliar products independent of a PACE label (*p* = 0.04) (2.9 ± 1.7 vs. 2.3 ± 1.5 respectively). Less health focused participants did not have higher consumption for familiar products in comparison to unfamiliar products independent of a PACE label (*p* = 0.10). 

The overall model with PACE labelling and physical activity as fixed factors and prospective consumption as the dependent variable was not statistically significant (*p* = 0.13). Furthermore, physically inactive participants had significantly higher levels of prospective consumption for familiar products in comparison to unfamiliar products independent of a PACE label (*p* = 0.03) (2.8 ± 1.2 vs. 2.0 ± 1.0 respectively). Physically active participants also had higher prospective consumption for familiar products in comparison to unfamiliar products (*p* = 0.04) (3.4 ± 2.0 vs. 2.7 ± 1.9 respectively). 

### 3.4. Consumption

For all products, the average total consumption was 8.5 g ± 7.7. Independent of a PACE label, consumption was higher for familiar products in comparison to unfamiliar products (*p* = 0.05) (10.6 g ± 8.2 vs. 8.3 g ± 8.1 respectively). The overall model with PACE labelling and health focus as fixed factors and consumption as the dependent variable was not statistically significant (*p* = 0.42). More health focused participants had higher levels of consumption for familiar products in comparison to unfamiliar products independent of a PACE label (*p* = 0.05) (10.1 ± 7.5 vs. 7.3 ± 7.4). Consumption for less health focused participants was not significant (*p* = 0.41).

The overall model with PACE labelling and physical activity as fixed factors and consumption as the dependent variable was not statistically significant (*p* = 0.73). For both physically active and inactive participants, the results for consumption between familiar and unfamiliar products were not significant independent of a PACE label (*p* = 0.18 and *p* = 0.09 respectively). Table 4 below displays the participant’s values (mean (SD)) for liking, prospective consumption and consumption for PACE and no PACE labels for both familiar and unfamiliar products. 

### 3.5. Association between Health Focus and Physical Activity 

To examine the potential association between participants being health focused and physically active, a Chi-Square test was performed. A statistically significant association between being more health focused and physically active was found (*p* = 0.05). Of the 97 participants, 48 participants were classed as both more health focused and physically active.

## 4. Discussion

The PACE label was found to significantly decrease liking among more health focused individuals but not among less health focused individuals which is consistent with previous findings [40]. Past research suggests that more health focused consumers are more inclined to look at nutrition related FOP labelling than less health focused consumers [40] Therefore, it can be suggested that in the present study, more health focused participants placed more emphasis on the PACE label than less health focused participants. It is unlikely that less health focused consumers did not understand the PACE label as symbolic labels are easy to understand [30,32,33] and liked by consumers [8]. Previous research has found that less health focused individuals have a poorer diet quality and show a lower engagement in healthy behaviours [37,38,39]. Which is in line with the findings of the present study. Recall that less health focused consumers had a higher BMI and were less physically active. Research has also found that although some less health focused consumers are aware that unhealthy eating is a major contributor to diseases, this understanding is not reflected in their actual food choices [47]. 

In terms of previous research, studies that have focused on PACE labelling have used participant groups from the general population rather than analyzing more and less health focused participants specifically [8,10,30]. The participant groups range in ages, genders, professions and backgrounds in all of the studies [8,10,30]. Few studies have assessed health focus in conjunction with labelling and caloric intake [28] and food decision making [47]. 

It can be argued that less health focused individuals are more in need of an effective labelling strategy than more health focused individuals as their lifestyle and overall wellness tend to be poorer [37,38]. As the PACE label was not effective among less health focused individuals to decrease discretionary snack food consumption, an alternative strategy should be considered. Even though the PACE label shows some potential in previous studies to change food choices and reduce calorie consumption in specific scenarios among the general population (not more and less health focused people specifically) [8,10,30], this study illustrates that it is not effective in the general population. 

To be an effective label overall, the PACE label must be effective throughout the general population, not just the segmented groups. To maximise the effectiveness of the PACE label, a mixed approach should be considered to make the PACE label more successful in reducing the consumption of discretionary foods. One strategy would be to combine PACE labelling with education and make less health focused consumers, more health focused. However, this approach is problematic, because various studies have suggested that it is very difficult to convert less health focused people into more health focused consumers [48,49]. Changing health related behaviours, is often a complicated process as it requires two separate processes involving motivation and volition [50]. These processes are based on the social-cognition models of health behaviour change [50]. To successfully change health related behaviours, the individual must have motivation to change [50]. This change must then be planned, initiated, and maintained and self-regulation plays a critical role in this [50]. For less health focused individuals, there may be no desire to become more health focused or no motivation to change this behaviour. They may believe that obesity is not a serious or relevant issue for them [50], it is too difficult to eat healthily, or that the benefit of healthy eating does not outweigh the barriers or negative consequences (e.g., lack of taste) [47]. This lack of motivation therefore makes it difficult to successfully change from being less health focused to more health focused. Few studies examining the PACE label have also assessed the participant’s attitudes towards health, healthy eating and their health literacy [8,30]. One method used to assess participant’s health literacy was the validated Newest Vital Sign method and their health literacy was deemed “adequate” if participants answered at least four out of six questions correctly [30]. In terms of health literacy, only 55% had “adequate” health literacy and 77% of participants stated that they had eaten fast-food within the past week and that 57% consider the “healthiness” of fast food menu items while ordering [30]. 

An alternative approach is to make health related messages more personally relevant to consumers who are not focused on health per se [51]. The Elaboration Likelihood Model suggests that people are more likely to process information thoughtfully when an issue is personally relevant [52]. For more health focused consumers specifically, previous studies have found that this subgroup can find health related messages more personally relevant, and may focus more on them and think more systematically about the recommendations included in the message, than less health focused consumers [53]. This is due to a positive relationship between more health focused individuals and adaptive coping (which implies a motivation to comply with recommendations and to adopt lasting changes in an individual’s health-related behaviours [54,55]) [56]. Less health focused consumers are more focused on short term gains such as taste and price [47].

When making food choices, consumers are often confronted with a dilemma of self-control [57,58]. Consumers often have to compromise between a short-term hedonic goal of tasty food intake and a long-term goal of healthy nutrition. For less health focused individuals, this desire to eat tasty food and indulge in tasty foods which are high in fat, sugar and salt, often contradicts the desire to eat healthily [47]. This is underpinned by the belief that unhealthy food tastes better than healthy food [47]. It then becomes difficult to overcome this belief and increase diet quality with FOP labelling (such as a PACE label) alone [47]. Therefore, FOP labelling needs to counter these beliefs by emphasizing the good taste and quality of healthy food products. Several studies have shown that labels which mention taste [42,59], country of origin [60] and sustainable practices [61,62] can all influence consumers’ perception and in some cases, improve intake of a wide variety of products. However there has been no research conducted that has explored combining these labelling approaches with PACE labelling. It is therefore recommended that on healthy products, the PACE label is used in combination with labels which can improve product perception. The challenge of how to decrease the consumption of unhealthy foods, however, still remains. As the food industry wants to drive sales of their products, it seems illogical for them to say to consumers that their product is not tasty. 

Another strategy is to work with the food industry and combine PACE labelling with slight product reformulation. A slight change in the nutritional profile (such as a reduction in sugar, salt or fat) of the product combined with PACE labelling could be effective in reducing the intake of unhealthy ingredients while consumers eat discretionary foods. However major product reformulation is sometimes not popular with consumers as they feel their choice is being taken away [21].

### Limitations

There are several limitations to the present study. This study had a relatively small sample size of 97 participants. However, power analyses, based on a linear multiple regression with an assumed small to medium effect size (f^2^ = 0.15) [34], suggest that a total sample size of *n* = 71 provides enough power to detect differences in liking and/or consumption. A second limitation to this study is that the controlled laboratory-like environment where the participants were tested may not be a true reflection of actual consumer behaviour in a supermarket or restaurant. This limitation was also observed by previous research [31,63,64]. A further limitation to the study is that participants’ cultural background was not factored into the study design as this may have affected their familiarity to the samples. This would be interesting to explore this further, perhaps in future studies. An additional limitation to the study is that a large portion of the participants were female (35% males and 65% female) which may have impacted on the data. Women have been found to be more health focused than males and tend to pay attention to FOP labelling more often [40,65]. Therefore, if the ratio of male and female participants is skewed, this may result in an increased power of the PACE label which would not be true outside of a laboratory setting.

## 5. Conclusions

Although symbolic labelling has been found to effectively decrease calorie consumption and is preferred by consumers over other forms of FOP labelling, in this study, PACE labelling was not effective in reducing the liking, prospective consumption or consumption of discretionary snack foods for less health focused individuals. The PACE label was found be effective in reducing liking for more health focused individuals however for less health focused individuals or a wider population, who are in need of an effective labelling strategy the most, it was not effective overall. For physically active and physically inactive individuals, there was found to be no significant effects of a PACE label. As the PACE label was found to not be effective among less health focused people or for the overall sample, it may not be an effective strategy to reduce the liking or consumption of discretionary snack foods for the general population.

## Figures and Tables

**Table 1:** Samples and their corresponding blinding codes.

Blinding Code	Sample
843	Control: Sakata Seaweed Rice Cracker
157	PACE: Arnott’s Nacho Cheese Shapes
825	Arnott’s Nacho Cheese Shapes
125	PACE: Want Want Cheese Rice Cracker
427	Want Want Cheese Rice Cracker

**Table 2:** Statements from the General Health Interest Scale [41].

I am very particular about the healthiness of food
I always follow a healthy and balanced diet
It is important for me that my diet is low in fat
It is important to me that my daily diet contains a lot of vitamins and minerals
I eat what I like and do not worry about healthiness of food ^R^
The healthiness of food has little impact on my food choice ^R^
The healthiness of snacks makes no difference to me ^R^
I do not avoid any foods, even if they may raise my cholesterol ^R^

^R^ Statements were recoded for calculation; 7-point Likert-type summated scale was used.

**Table 3:** Descriptive statistics of participants.

	Less Health Focused ^2^ (*n* = 40) (mean ± SD)	More Health Focused ^2^ (*n* = 57) (mean ± SD)
Male:Female *	43%:58%	30%:70%
Age (years)	34 ± 12	36 ± 14
Height (cm)	164 ± 27	165 ± 17
Weight * (kgs)	88 ± 19	67 ± 13
BMI *	32 ± 7	25 ± 5
Physically active ^1,^*	58%	32%
Physically inactive ^1^	42%	68%

^1^ Physical activity calculated using the IPAQ formula [44,45]; ^2^ Health focus calculated using the General Health Interest Scale [41]. * Statistically significant difference between less and more health focused *p* < 0.05.

**Table 4:** Mean (SD) values of liking, prospective consumption and consumption for PACE and no PACE label for familiar and unfamiliar products.

	Liking	Prospective Consumption	Consumption
	Mean (SD)	Mean (SD)	Mean (SD)
**Familiar Product (Arnott’s Nacho Cheese Shapes)**			
PACE	6.50 (1.8)	3.18 (2.1)	10.01 (8.5)
No PACE	6.85 (1.5)	3.34 (1.7)	11.17 (7.8)
**Unfamiliar Product (Want Want Cheese Rice Crackers)**			
PACE	5.24 (2.5)	2.42 (1.5)	8.17 (8.3)
No PACE	5.86 (2.2)	2.79 (2.1)	8.47 (7.9)
